# Supplementary material for: A glutamate receptor C-tail recruits CaMKII to suppress retrograde homeostatic signaling
Source: Nat Commun. 2022 Dec 10;13:7656. doi: 10.1038/s41467-022-35417-9 (PMC9741633; doi:10.1038/s41467-022-35417-9)
Supplement: Supplementary file 1 — Supplementary Information [file 41467_2022_35417_MOESM1_ESM.pdf]

Supplementary Information

A glutamate receptor C-tail recruits CaMKII to suppress retrograde homeostatic signaling

Perry et al.

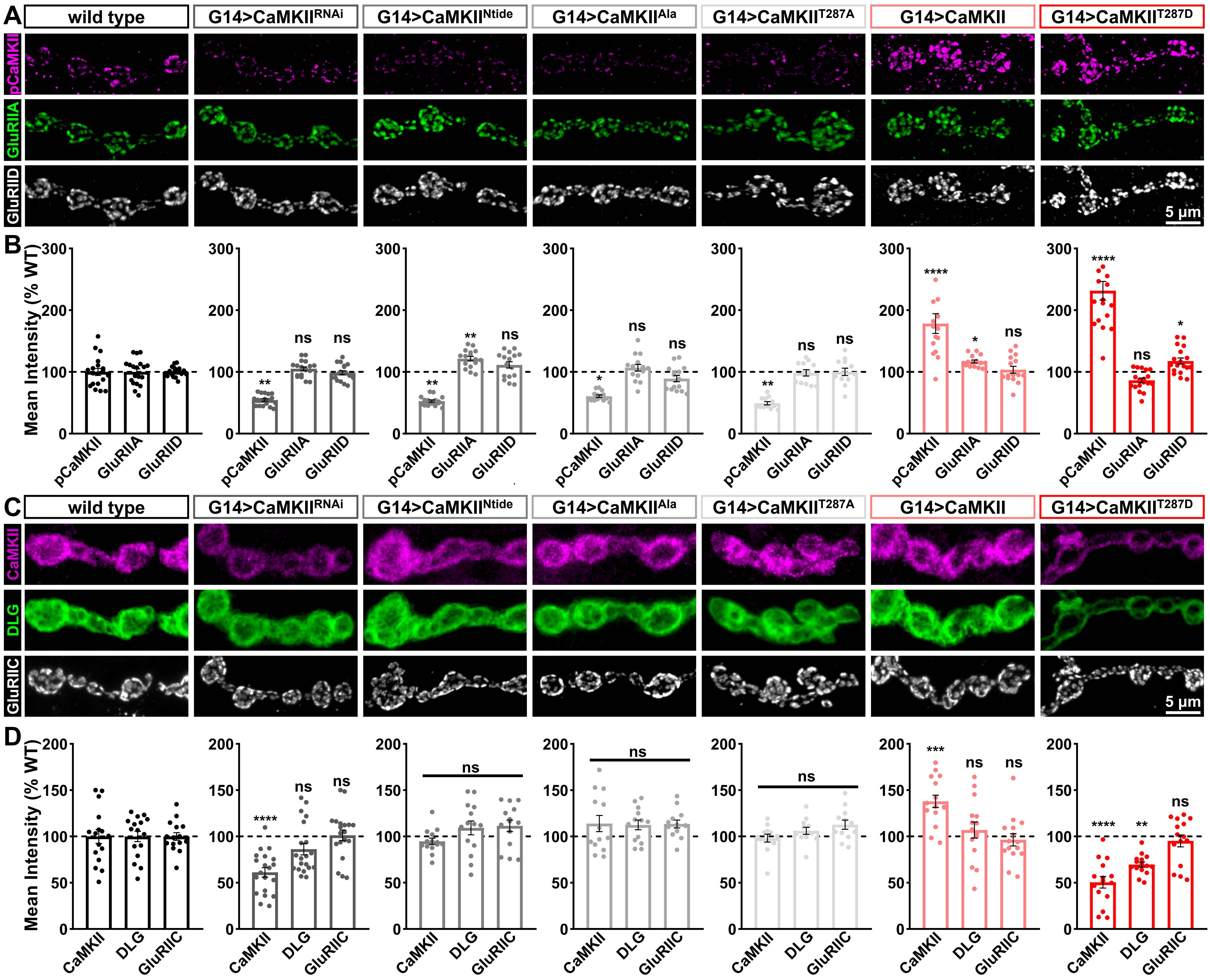

A
wild type
G14>CaMKII^RNAi
G14>CaMKII^Ntide
G14>CaMKII^Ala
G14>CaMKII^T287A
G14>CaMKII
G14>CaMKII^T287D
pCaMKII
GluRIIA
GluRIID
5 μm
B
Mean Intensity (% WT)
pCaMKII
GluRIIA
GluRIID
ns
*
**
****
C
CaMKII
DLG
GluRIIC
D
***

Supplementary Figure S1: Validation of the anti-pCaMKII and -CaMKII antibodies. (A) Representative images of NMJ boutons immunostained with anti-pCaMKII, -GluRIIA and -GluRIID antibodies in the indicated genotypes: G14>CaMKII^{RNAi} (postsynaptic CaMKII knock down: *w*;*G14-GAL4*/+;*UAS-CaMKII^{RNAi}*/+), G14>CaMKII^{Ntide} (peptide inhibitor of CaMKII: *w*;*G14-GAL4*/*USA-CaMKII^{Ntide}*;+), G14>CaMKII^{Ala} (peptide inhibitor of CaMKII: *w*;*G14-GAL4*/*UAS-CaMKII^{Ala}*;+), G14>CaMKII^{T287A} (constitutively inactive CaMKII: *w*;*G14-GAL4*/*UAS-CaMKII^{T287A}*;+); G14>CaMKII (CaMKII overexpression: *w*;*G14-GAL4*/+;*UAS-CaMKII*/+), G14>CaMKII^{T287D} (constitutively active CaMKII: *w*;*G14-GAL4*/*UAS-CaMKII^{T287D}*). Experiments were repeated three times independently with similar results. **(B)** Quantification of mean fluorescence intensity of pCaMKII, GluRIIA and GluRIID puncta normalized to wild-type values. (wild type: n=19; G14>CaMKII^{RNAi}, n=18, p=0.0012 for pCaMKII, p=0.8801 for GluRIIA, p=0.9997 for GluRIID; G14>CaMKII^{Ntide}: n=16, p=0.0011 for pCaMKII, p=0.0013 for GluRIIA, p=0.2781 for GluRIID; G14>CaMKII^{Ala}: n=15, p=0.0112 for pCaMKII, p=0.7049 for GluRIIA, p=0.3413 for GluRIID; G14>CaMKII^{T287A}: n=12, p=0.0014 for pCaMKII, p=0.9996 for GluRIIA, p>0.9999 for GluRIID; G14>CaMKII: n=15, p<0.0001 for pCaMKII, p=0.0190 for GluRIIA, p=0.9871 for GluRIID; G14>CaMKII^{T287D}: n=18, p<0.0001 for pCaMKII, p=0.0644 for GluRIIA, p=0.0218 for GluRIID). Repeated measures One-way ANOVA with Dunnett's multiple comparisons test with a significance value of 0.05; p-value was adjusted for multiple comparison. GluRIIA levels are unexpectedly increased in G14>CaMKII^{Ntide}, which may be caused by developmental changes in the postsynaptic apparatus due to strong and constitutive inhibition of CaMKII activity. **(C)** Representative images of NMJ boutons immunostained with anti-CaMKII, -DLG and -GluRIIC antibodies in the same genotypes in (A). Experiments were repeated four times independently with similar results. **(D)** Quantification of mean fluorescence intensity of CaMKII, DLG, and GluRIIC puncta normalized to wild-type values. (wild type: n=16; G14>CaMKII^{RNAi}, n=20, p<0.0001 for CaMKII, p=0.3428 for DLG, p=0.9997 for GluRIIC; G14>CaMKII^{Ntide}: n=14, p=0.9814 for CaMKII, p=0.7943 for DLG, p=0.5294 for GluRIIC; G14>CaMKII^{Ala}: n=13, p=0.4469 for CaMKII, p=0.5515 for DLG, p=0.3793 for GluRIIC; G14>CaMKII^{T287A}: n=12, p=0.9997 for CaMKII, p=0.9689 for DLG, p=0.4644 for GluRIIC; G14>CaMKII: n=15, p=0.0002 for CaMKII, p=0.9259 for DLG, p=0.9946 for GluRIIC; G14>CaMKII^{T287D}: n=15, p<0.0001 for CaMKII, p=0.0033 for DLG, p=0.9785 for GluRIIC). Repeated measures One-way ANOVA with Dunnett's multiple comparisons test with a significance value of 0.05. p-value was adjusted for multiple comparison. Error bars indicate $\pm$SEM. Asterisks indicate statistical significance using One-way ANOVA: *P<0.05, **P<0.01, ***P<0.001, ****P<0.0001; ns, not significant. n values indicate biologically independent NMJs. Source data are provided as a Source Data file.

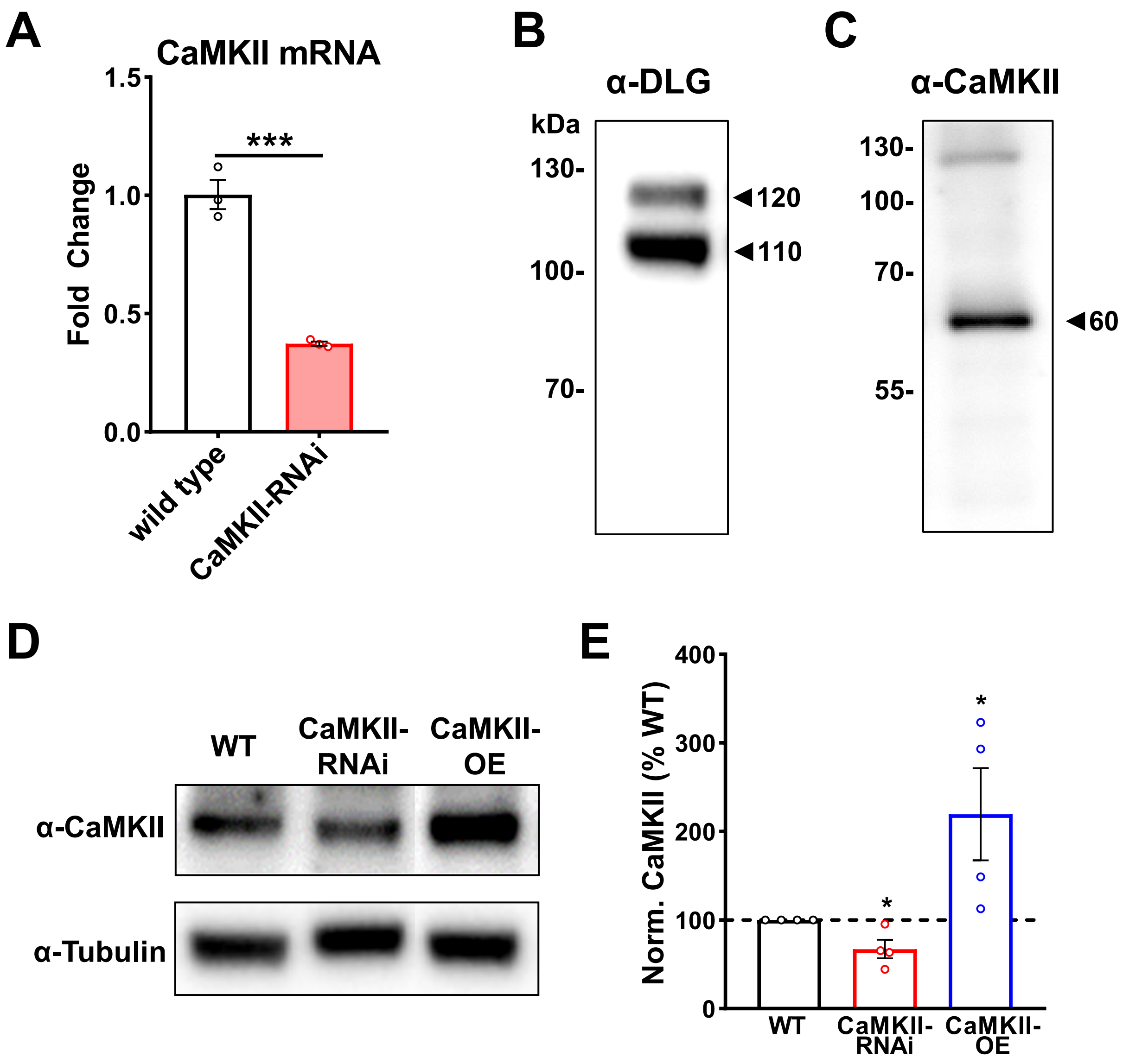

A
CaMKII mRNA
1.5
1.0
0.5
0.0
Fold Change
***
wild type
CaMKII-RNAi
B
α-DLG
kDa
130-
100-
70-
◀120
◀110
C
α-CaMKII
130-
100-
70-
55-
◀60
D
WT
CaMKII-RNAi
CaMKII-OE
α-CaMKII
α-Tubulin
E
Norm. CaMKII (% WT)
400
300
200
100
0
*
*
WT
CaMKII-RNAi
CaMKII-OE

Supplementary Figure S2: Muscle CaMKII is expressed as a single 60 kDa band on immunoblots. (A) qPCR analysis showing an ~60% reduction in CaMKII mRNA levels following muscle-specific RNAi knock-down. (wild type: n=3; CaMKII-RNAi, n=3, p=0.0005, unpaired, two-tailed t-test) **(B)** Anti-DLG immunoblot from wild-type larval muscle lysates. Note the two isoforms of DLG are clearly observed, corresponding to DLGA (~110 kDa) and DLGS97 (~120 kDa). **(C)** Anti-CaMKII immunoblot from wild-type muscle lysates. Note that *Drosophila* CaMKII runs as single band at ~60 kDa, as expected. **(D)** Anti-CaMKII immunoblots from muscle lysates of wild type, CaMKII-RNAi (G14>$CaMKII^{RNAi}$), and CaMKII-OE (G14>CaMKII). Anti-β-tubulin blots are shown below as loading controls. **(E)** The quantification shows CaMKII/β-tubulin intensity ratios (normalized to wild type controls) averaged from 4 replicates per genotype (12 larvae per genotype). (wild type: n=4; CaMKII-RNAi, n=4, p=0.0286; CaMKII-OE, n=4, p=0.0286, paired, two-tailed t-test). Error bars indicate ±SEM. Asterisks indicate statistical significance using One-way ANOVA: *P<0.05, ***P<0.001. Source data are provided as a Source Data file.

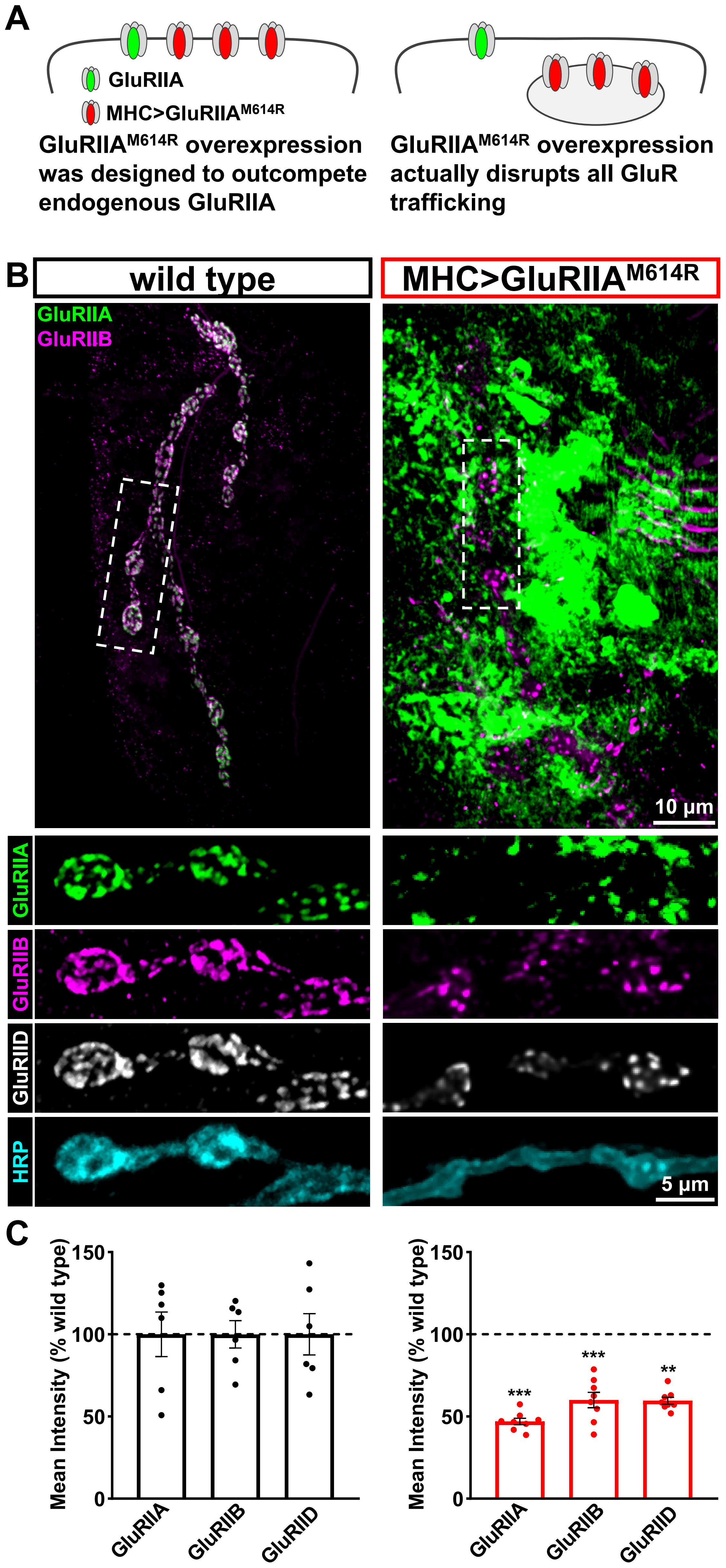
A
GluRIIA
MHC>GluRIIAM614R
GluRIIAM614R overexpression was designed to outcompete endogenous GluRIIA
GluRIIAM614R overexpression actually disrupts all GluR trafficking
B
wild type
MHC>GluRIIAM614R
GluRIIA
GluRIIB
10 µm
GluRIIA
GluRIIB
GluRIID
HRP
5 µm
C
Mean Intensity (% wild type)
150
100
50
0
GluRIIA
GluRIIB
GluRIID
***
***
**

Supplementary Figure S3: Postsynaptic *GluRIIA^{M614R}* overexpression disrupts GluR trafficking. (A) Schematic illustrating the expected and actual GluRA and GluRB receptor distributions at postsynaptic compartments in wild type and postsynaptic *GluRIIA^{M614R}* overexpression. **(B)** Representative images of muscle 4 NMJs immunostained with anti-GluRIIA, -GluRIIB, -GluRIID and -HRP antibodies in wild type or postsynaptic overexpression of the *GluRIIA^{M614R}* transgene: MHC>GluRIIA^{M614R} (*w*;+;*MHC-GAL4*/*UAS-GluRIIA^{M614R}*). Note that all GluRs are reduced at postsynaptic compartments in MHC>GluRIIA^{M614R}, with high levels of GluRIIA apparently accumulating in intracellular muscle compartments. Experiments were repeated three times independently with similar results. **(C)** Quantification of mean fluorescence intensities of GluRIIA, GluRIIB and GluRIID subunits at NMJs normalized to wild-type values (wild type: n=6; MHC>CaMKII^{M614R}: n=8, p=0.0007 for GluRIIA, p=0.0007 for GluRIIB, p=0.0032 for GluRIID, unpaired, two-tailed t-test, with a significance value of 0.05). Error bars indicate ±SEM. **P<0.01, ***P<0.001. n values indicate biologically independent NMJs. Source data are provided as a Source Data file.

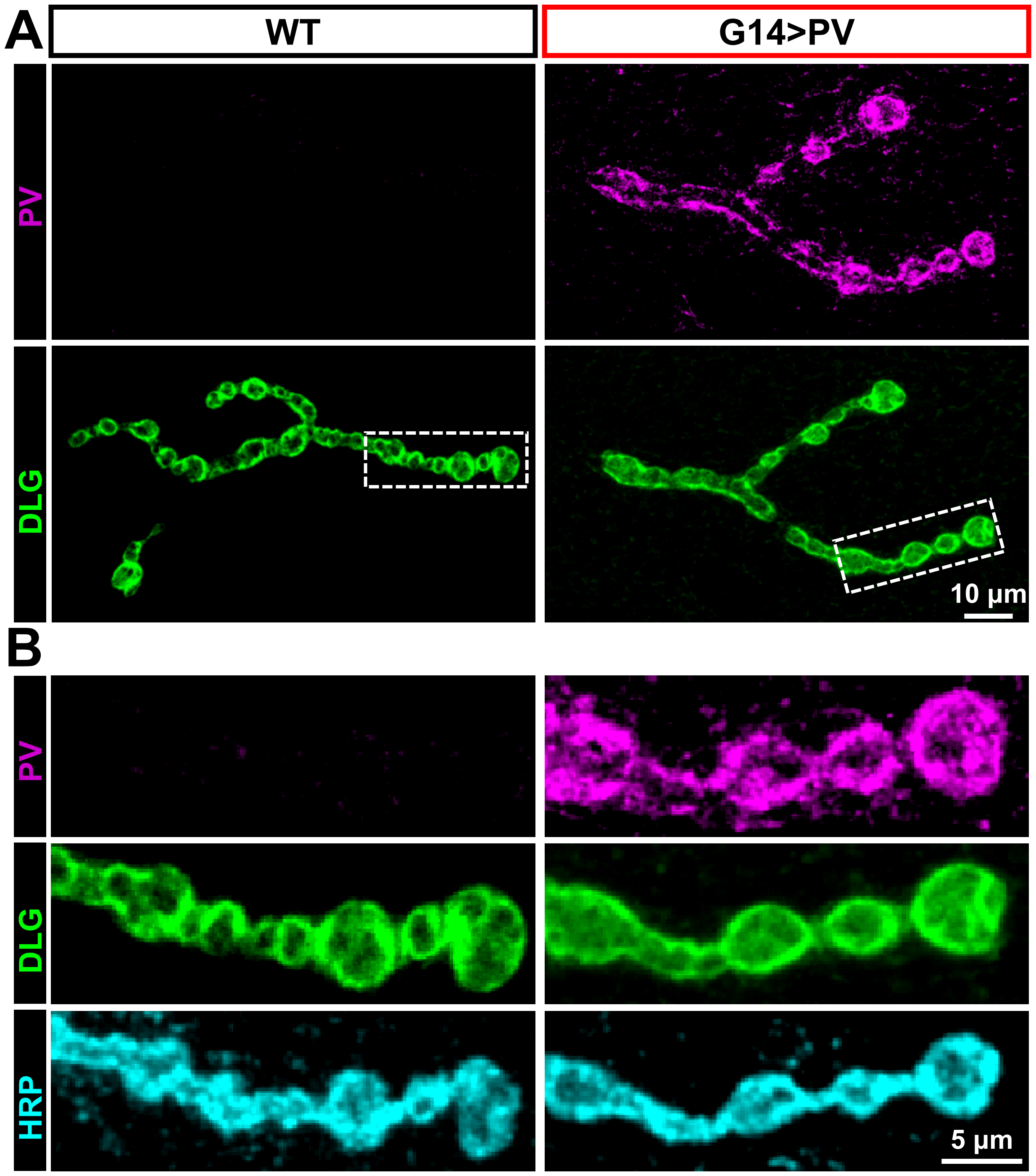
A
WT
G14>PV
PV
DLG
10 µm
B
PV
DLG
HRP
5 µm

Supplementary Figure S4: Parvalbumin localizes to postsynaptic compartments when expressed in muscle. (A) Representative images of NMJs immunostained with anti-parvalbumin (PV) and -DLG in wild type and postsynaptic expression of PV: G14>PV (*w*;*G14-GAL4*/+;*UAS-PV*/+). **(B)** Images of individual boutons stained with anti-PV, -DLG and -HRP in the indicated genotypes showing that PV largely overlaps with the postsynaptic density marker DLG. (**A, B**) Experiments were repeated four times independently with similar results.

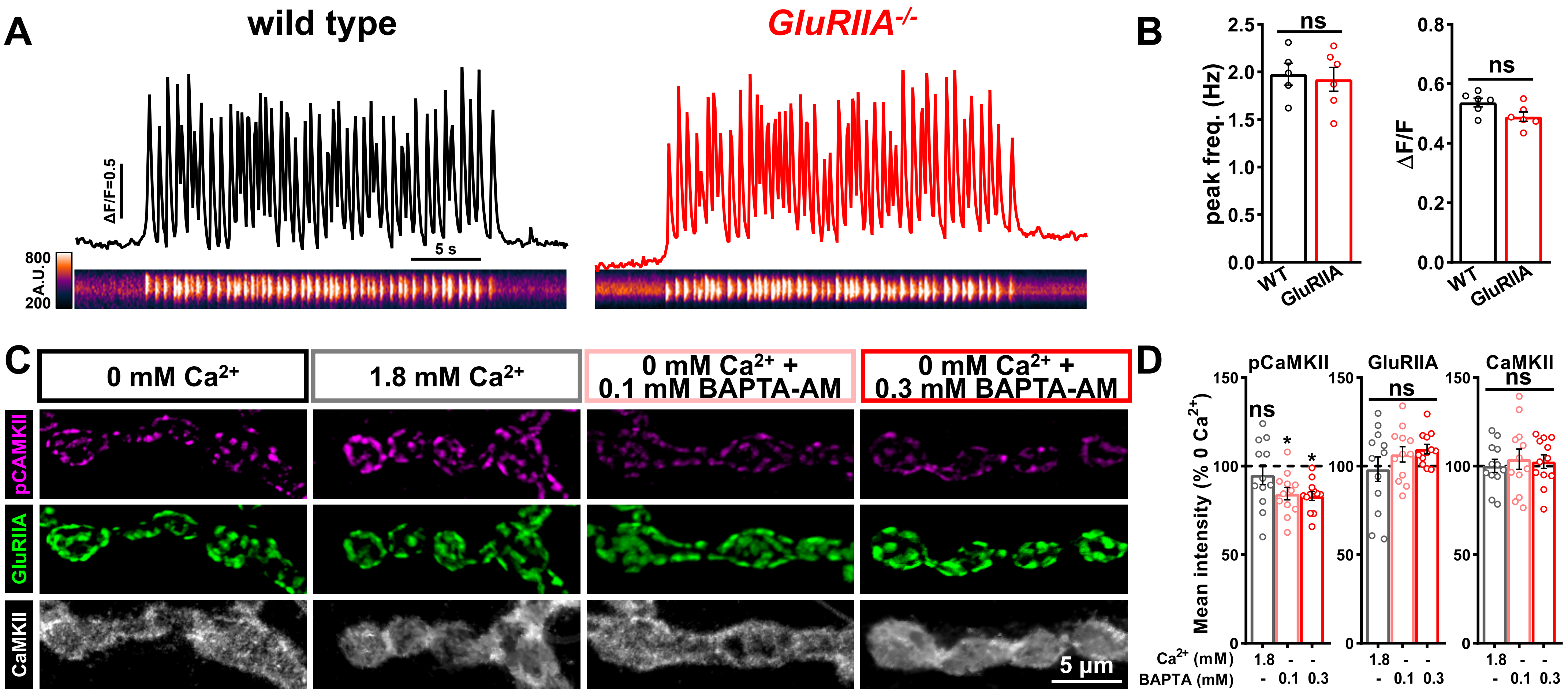

A
wild type
GluRIIA-/-
ΔF/F=0.5
5 s
800
A.U.
200
B
peak freq. (Hz)
2.5
2.0
1.5
1.0
0.5
0.0
ns
WT
GluRIIA
ΔF/F
0.8
0.6
0.4
0.2
0.0
ns
WT
GluRIIA
C
0 mM Ca2+
1.8 mM Ca2+
0 mM Ca2+ + 0.1 mM BAPTA-AM
0 mM Ca2+ + 0.3 mM BAPTA-AM
pCAMKII
GluRIIA
CaMKII
5 µm
D
pCaMKII
GluRIIA
CaMKII
Mean intensity (% 0 Ca2+)
150
100
50
0
ns
*
*
ns
ns
Ca2+ (mM) 1.8 - -
BAPTA (mM) - 0.1 0.3

Supplementary Figure S5: Evidence that pCaMKII levels are not significantly controlled by patterns of activity of intracellular Ca^{2+} levels: (A) Patterns of SynapGCaMP8f Ca^{2+} transients imaged in semi-intact preparations at tonic Ib boutons of muscle 6 in wild type and *GluRIIA* mutants. Similar firing patterns are observed in both genotypes. **(B)** Quantification of firing frequency and amplitude of Ca^{2+} transients imaged, with no significant differences observed between wild type and *GluRIIA* mutants. (wild type: n=6; $GluRIIA^{-/-}$, n=6, p=0.7502 for peak frequency, p=0.0514 for ΔF/F, unpaired, two-tailed t-test, with a significance value of 0.05). **(C)** Representative images of wild-type NMJs immunostained with anti-pCaMKII, -GluRIIA, and CaMKII in 0 Ca^{2+} saline, saline supplemented with 1.8 mM Ca^{2+}, or 0 Ca^{2+} saline supplemented with 100 or 300 µM BAPTA-AM. Experiments were repeated three times independently with similar results. A small but significant reduction in the pCaMKII signal is observed only after BAPTA-AM application. **(D)** Quantification of mean fluorescence intensity levels in the indicated conditions normalized to the 0 Ca^{2+} control. (0 mM Ca^{2+}: n=12; 1.8 mM Ca^{2+}, n=12, p=0.7379 for pCaMKII, p=0.7147 for GluRIIA, p>0.9999 for CaMKII; 0 mM Ca^{2+}+0.1 mM BAPTA: n=12, p=0.0280 for pCaMKII, p=0.9912 for GluRIIA, p=0.8353 for CaMKII; 0 mM Ca^{2+}+0.3 mM BAPTA: n=12, p=0.0161 for pCaMKII, p=0.4475 for GluRIIA, p=0.9495 for CaMKII). Repeated measures One-way ANOVA with Dunnett's multiple comparisons test with a significance value of 0.05. p value adjusted for multiple comparison. Error bars indicate ±SEM. Asterisks indicate statistical significance using t-test or One-way ANOVA: *P<0.05, ns = not significant. n values indicate biologically independent NMJs. Absolute values for normalized data are summarized in Table S1. Source data are provided as a Source Data file.

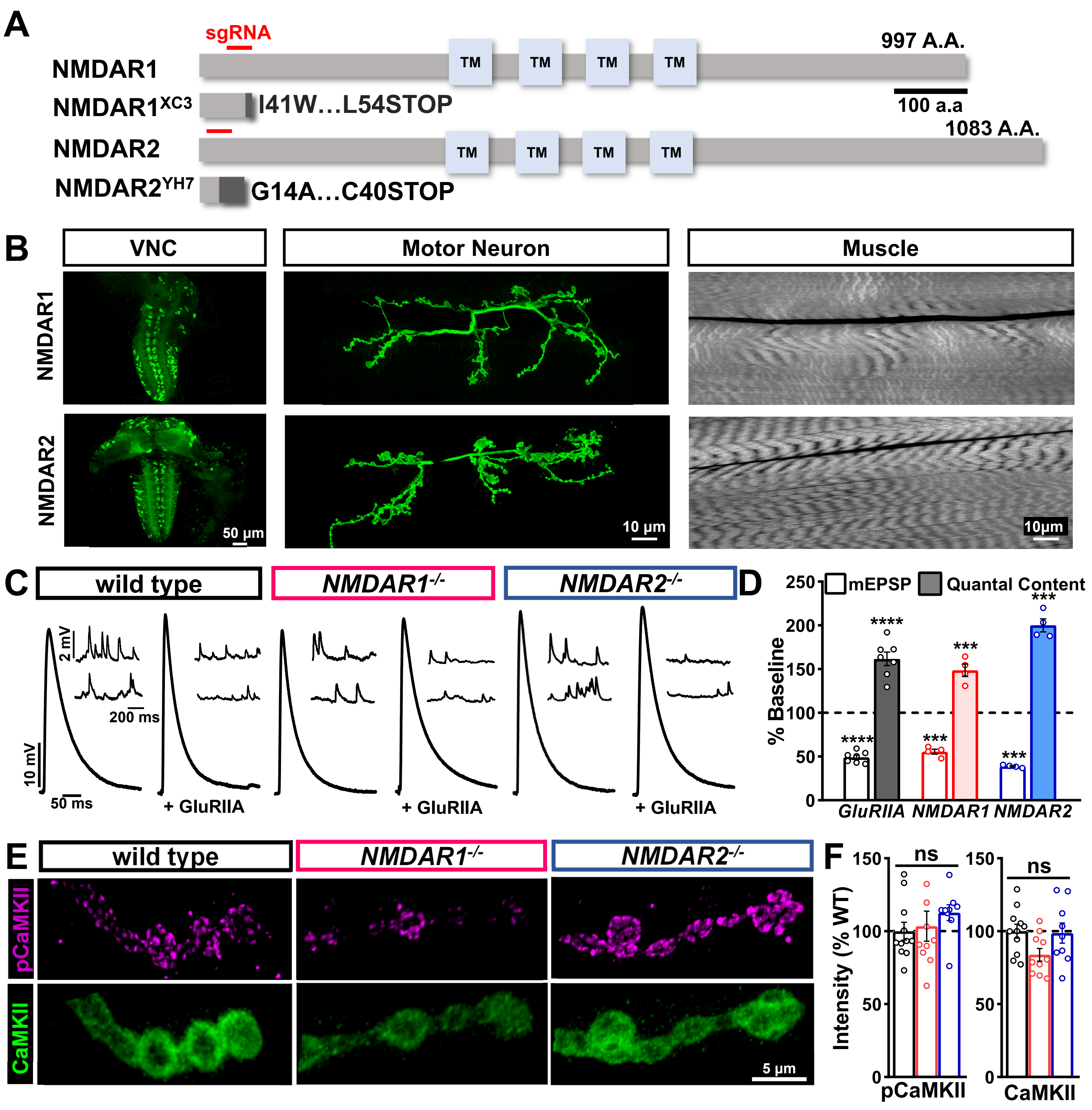
A
sgRNA
NMDAR1
997 A.A.
TM
100 a.a
NMDAR1XC3
I41W…L54STOP
NMDAR2
1083 A.A.
NMDAR2YH7
G14A…C40STOP
B
VNC
Motor Neuron
Muscle
NMDAR1
NMDAR2
50 µm
10 µm
10µm
C
wild type
NMDAR1-/-
NMDAR2-/-
2 mV
200 ms
10 mV
50 ms
+ GluRIIA
D
mEPSP
Quantal Content
% Baseline
GluRIIA
NMDAR1
NMDAR2
E
pCaMKII
CaMKII
5 µm
F
Intensity (% WT)
ns
pCaMKII
CaMKII

Supplementary Figure S6: *Drosophila* NMDA receptors do not influence CaMKII activity or PHP expression. (A) Schematics of the two NMDA receptors encoded in the *Drosophila* genome, NMDAR1 and NMDAR2. The amino acids targeted by the sgRNA for CRISPR/Cas9 mutagenesis are shown in red. Two NMDA mutant alleles generated are illustrated below, with frameshift mutations induced, encoding a short series of random amino acids (illustrated in black) and early termination at a stop codon. **(B)** Larval expression analysis of *T2A-GAL4* knock-ins of *NMDAR1* crossed to GFP reporters (*w*;*UAS-CD4::tdGFP*/+;*NMDAR1-T2A-GAL4*/+) and *NMDAR2* (*NMDAR2-T2A-GAL4*/+;*UAS-CD4::tdGFP*/+;+). Experiments were repeated three times independently with similar results. Note that both receptors are expressed broadly in the ventral nerve cord (VNC) and in motor neurons but are not expressed in muscle. **(C)** Representative electrophysiological traces from wild-type and NMDAR-mutant NMJs at baseline and in combination with *GluRIIA* mutations (*w;GluRIIA^{SP16};NMDAR1^{XC3}* and *w,NMDAR2^{YH7};GluRIIA^{SP16}*). **(D)** Quantification of mEPSP and quantal content values in the indicated genotypes normalized to baseline values (without *GluRIIA* mutant alleles). (wild type: n=10; GluRIIA$^{-/-}$, n=7, p<0.0001 for mEPSP, p<0.0001 for QC; NMDAR1$^{-/-}$, n=7; NMDAR1$^{-/-}$+GluRIIA$^{-/-}$, n=4, p=0.0001 for mEPSP, p=0.0010 for QC; NMDAR2$^{-/-}$, n=4; NMDAR2$^{-/-}$+GluRIIA$^{-/-}$: n=4, p=0.0001 for mEPSP, p=0.0004 for QC, unpaired, two-tailed t-test, with a significance value of 0.05). Robust PHP expression is observed despite loss of *NMDAR1* or *NMDAR2*. **(E)** Representative immunostaining of pCaMKII and CaMKII at NMJs in the indicated genotypes. Experiments were repeated three times independently with similar results. **(F)** Quantification of pCaMKII and CaMKII mean intensity normalized as a percentage of wild-type values in the indicated genotypes. (wild type: n=11; NMDAR1$^{-/-}$: n=10, p=0.9262 for pCaMKII, p>0.0689 for CaMKII; NMDAR2$^{-/-}$: n=9, p=0.4086 for pCaMKII, p=0.9810 for CaMKII). Repeated measures One-way ANOVA with Dunnett's multiple comparisons test with a significance value of 0.05. p-value was adjusted for multiple comparison. No significant differences are observed. Error bars indicate ±SEM. Asterisks indicate statistical significance using t-test or One-way ANOVA: ***P<0.001, ****P<0.0001, ns, not significant. n values indicate biologically independent NMJs. Absolute values for normalized data are summarized in Table S1. Source data are provided as a Source Data file.

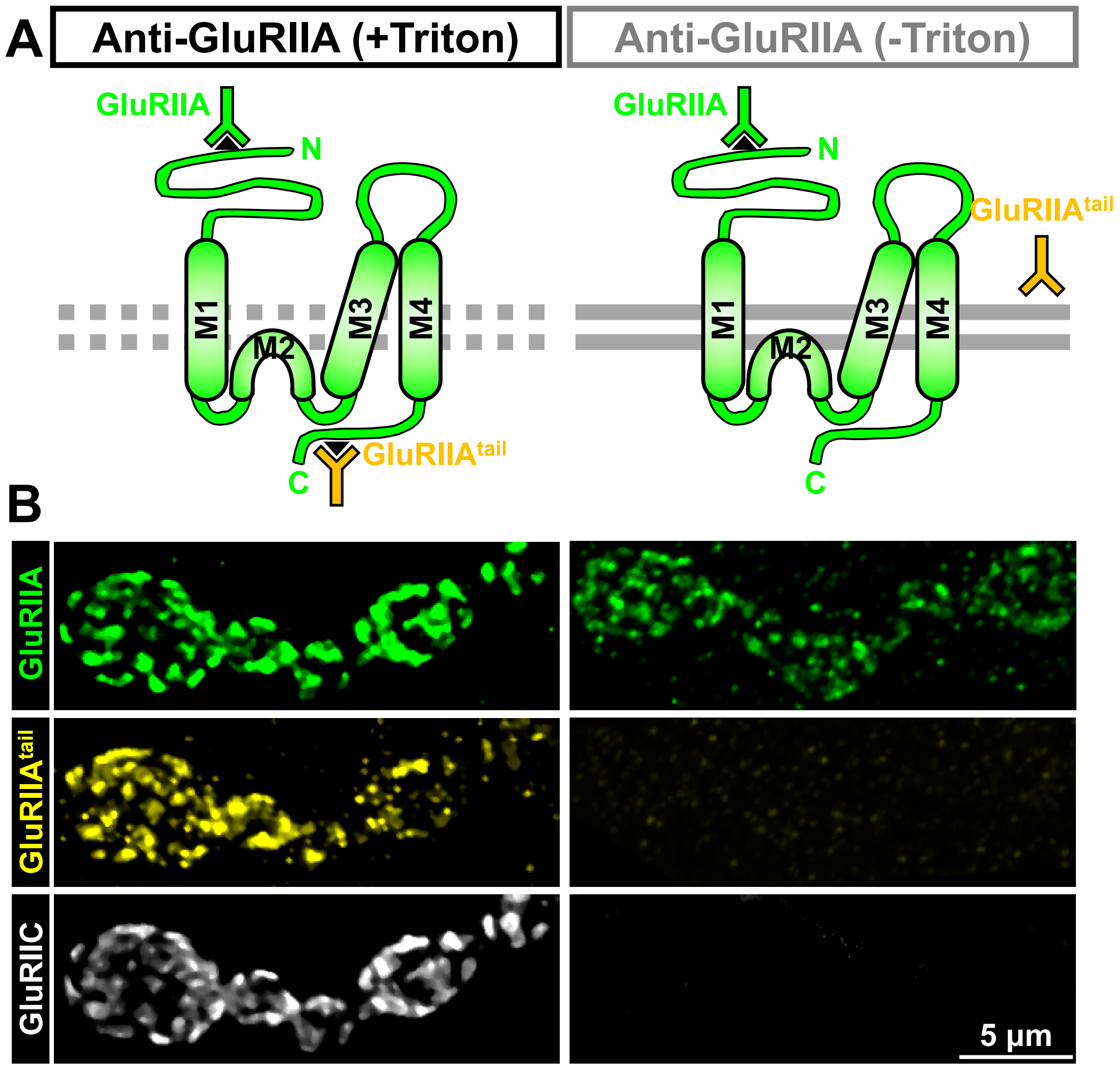
A
Anti-GluRIIA (+Triton)
Anti-GluRIIA (-Triton)
GluRIIA
N
M1
M2
M3
M4
C
GluRIIA^tail
B
GluRIIA
GluRIIA^tail
GluRIIC
5 µm

Supplementary Figure S7: Antigenic topology of the monoclonal GluRIIA antibody 8B4D2. (A) Schematic illustrating the membrane topology of the GluRIIA subunit and use of detergent (Triton) to determine the intracellular vs extracellular antigenic location of the monoclonal GluRIIA antibody 8B4D2. **(B)** Representative images of NMJ boutons immunostained with anti-GluRIIA, -GluRIIA^{tail}, and -GluRIIC antibodies. Experiments were repeated three times independently with similar results. The antigenic region of the anti-GluRIIA monoclonal antibody resides in an extracellular region of GluRIIA, while both anti-GluRIIA^{tail} and anti-GluRIIC antibodies recognize the intracellular C-tail of the relevant GluR subunit, as expected.

Supplementary Table 1: Absolute values for normalized data and additional statistical details. The figure and panel, genotype, and conditions are noted. Average values (with standard error of the mean noted in parentheses), data samples (n), and statistical significance tests and values are shown for all data.

Figure	Label	Genotype	mEPSP amplitude (mV)	EPSP amplitude (mV)	QC	mEPSP frequency (Hz)	Rinput (MΩ)	Resing potential (mV)	n	P Value (significance): mEPSP, EPSP, QC
1C	wild type	*w*^{1118}	1.035 (±0.045)	33.297 (±1.980)	32.125 (±1.100)	3.540 (±1.100)	9.50 (±0.307)	-69.172 (±1.707)	10	-
1C	*GluRIIA*$^{-/-}$	*w*;*GluRIIA*^{SP16}	0.463 (±0.027)	31.493 (±0.920)	69.123 (±2.271)	2.978 (±0.097)	11.50 (±0.268)	-71.986 (±1.611)	10	<0.0001 (****), 0.421 (ns), <0.0001 (****)
1C	G14>CaMKII^{T287D}	*w*;*G14-GAL4/+*; UAS-CaMKII^{T287D}/+	1.000 (±0.053)	34.777 (±1.962)	34.707 (±0.520)	3.883 (±1.962)	10.20 (±10.2)	-66.131 (±2.383)	10	-
1C	G14>CaMKII^{T287D} +GluRIIA$^{-/-}$	*w*;*G14-GAL4,GluRIIA*^{SP16}*/GluRIIA*^{SP16}; *UAS-CaMKII*^{T287D}*/+*	0.446 (±0.017)	16.213 (±0.365)	69.123 (±2.271)	3.065 (±0.186)	11.80 (±0.359)	-72.855 (±2.812)	10	<0.0001 (****), 0.0001 (****), <0.072 (ns)
3A	wild type	*w*^{1118}	1.035 (±0.083)	31.78 (±1.144)	32.46 (±3.376)	2.856 (±0.143)	??? (±)	??? (±)	8	-
3A	OK319>BoNT-C	*w*;*OK319-GAL4/+*; *UAS-BoNT-C/+*	0.0512 (±0.023)	1.43 (±0.29)	-	0.044 (±0.02)	7.63 (±0.46)	-74.59 (±3.40)	8	<0.0001 (****), 0.0001 (****)
4B-D	wild type	*w*^{1118}	1.018 (±1.018)	31.038 (±0.907)	31.503 (±2.253)	3.530 (±0.082)	9.41 (±0.608)	-69.179 (±2.174)	12	-
4B-D	*GluRIIA*$^{-/-}$	*w*;*GluRIIA*^{SP16}	0.433 (±0.025)	29.203 (±1.117)	69.670 (±4.543)	3.095 (±0.263)	10.91 (±0.56)	-71.927 (±1.532)	12	<0.0001 (****), 0.646 (ns), <0.0001 (****)
4B-D	*GluRIIA*^{Q615R}	*w*;*GluRIIA*^{Q615R}	0.983 (±0.027)	32.968 (±1.041)	33.579 (±0.884)	3.439 (±0.095)	13.00 (±0.507)	-67.363 (±1.966)	12	0.946 (ns), 0.607 (ns), 0.942 (ns)
4B-D	G14>PV	*w*;*G14-GAL4/+*; *UAS-PV/+*	1.020 (±0.053)	26.675 (±1.670)	26.442 (±1.533)	3.607 (±0.124)	9.08 (±0.542)	-67.656 (±2.418)	12	0.999 (ns), 0.041 (*), 0.437 (ns)
4B-D	G14>PV +*GluRIIA*^{Q615R}	*w*;*GluRIIA*^{Q615R}*,G14-GAL4/GluRIIA*^{Q615R}; *UAS-PV/+*	0.968 (±0.047)	31.983 (±1.030)	33.966 (±1.933)	3.517 (±0.119)	11.46 (±0.417)	-69.740 (±1.073)	13	0.812 (ns), 0.942 (ns), 0.893 (ns)
6B-D	wild type	*w*^{1118}	0.967 (±0.070)	29.844 (±1.016)	32.255 (±2.184)	3.049 (±0.108)	8.00 (±0.301)	-65.333 (±3.027)	11	-
6B-D	*GluRIIA-/-*	*w*;*GluRIIA*^{SP16}	0.475 (±0.028)	27.041 (±0.786)	58.885 (±3.936)	2.805 (±0.137)	11.00 (±0.333)	-66.874 (±3.300)	10	<0.0001 (****), 0.276 (ns), <0.0001 (****)
6B-D	*GluRIIA*$^{\Delta C20}$	*w*;*GluRIIA*$^{\Delta C20}$	1.045 (±0.053)	30.345 (±0.753)	30.108 (±1.956)	3.097 (±0.270)	9.30 (±0.307)	-69.720 (±2.299)	13	0.682 (ns), 0.992 (ns), 0.921 (ns)
6B-D	*GluRIIA*$^{\Delta C9}$	*w*;*GluRIIA*$^{\Delta C9}$	1.099 (±0.036)	32.151 (±0.738)	29.661 (±1.262)	2.884 (±0.422)	12.42 (±0.453)	-69.533 (±2.688)	14	0.230 (ns), 0.371 (ns), 0.852 (ns)
6B-D	*GluRIIA*$^{QR\Delta C19}$	*w*;*GluRIIA*$^{QR\Delta C19}$	1.000 (±0.069)	32.980 (±1.972)	34.678 (±2.997)	2.749 (±0.169)	12.90 (±0.414)	-69.449 (±3.077)	11	0.980 (ns), 0.176 (ns), 0.905 (ns)
8C-E	wild type	*w*^{1118}	1.029 (±0.051)	31.476 (±1.345)	31.421 (±2.213)	2.879 (±0.070)	12.00 (±0.381)	-66.272 (±3.715)	12	-
8C-E	*GluRIIA*$^{-/-}$	*w*;*GluRIIA*^{SP16}	0.038 (±0.051)	28.283 (±1.265)	63.079 (±4.979)	2.563 (±0.094)	8.90 (±0.433)	-66.272 (±2.485)	10	<0.0001 (****), 0.331 (ns), <0.0001 (****)
8C-E	G14>GluRIIB (IIA/IIB null)	*w*;*G14-GAL4,GluRIIA*^{SP22}*/Df(2L)cl*^{h4}; *UAS-GluRIIB/+*	0.471 (±0.031)	29.264 (±2.159)	65.435 (±6.023)	2.915 (±0.149)	11.90 (±0.563)	-62.019 (±2.631)	11	<0.0001 (****), 0.596 (ns), <0.0001 (****)
8C-E	G14>GluRIIB^{IIAtail} (IIA/IIB null)	w;*G14-GAL4, GluRIIA*^{SP22}*/Df(2L)cl*^{h4}; *UAS-GluRIIB*^{IIAtail}*/+*	0.466 (±0.033)	17.084 (±0.977)	38.857 (±3.815)	2.697 (±0.151)	10.91 (±0.312)	-72.750 (±3.831)	12	<0.0001 (****), <0.0001 (****), 0.490 (ns)
S3	wild type	*w*^{1118}	1.054 (±0.047)	30.539 (±2.015)	30.546 (±1.924)	3.027 (±0.089)	9.56 (±0. 772)	-65.493 (±4.576)	10	-
S3	MHC>GluRIIA^{M614R}	w;*UAS-GluRIIA*^{M614R}*/+*; *MHC-GAL4/+*	0.506 (±0.021)	33.400 (±0.785)	66.560 (±2.759)	2.697 (±0.151)	10.91 (±0.312)	-66.830 (±7.637)	6	<0.0001 (****), 0.573 (ns), <0.0001 (*****)
S6D	wild type	*w*^{1118}	1.120 (±0.062)	36.739 (±1.566)	33.256 (±1.537)	3.649 (±0.089)	15.20 (±0.742)	-72.744 (±3.105)	10	-
S6D	*GluRIIA*$^{-/-}$	*w*;*GluRIIA*^{SP16}	0.551 (±0.027)	29.308 (±1.566)	53.780 (±2.571)	3.026 (±0.212)	15.85 (±0.340)	-67.601 (±3.105)	7	<0.0001 (****), <0.05 (*), <0.0001 (*****)
S6D	*NMDAR1*$^{-/-}$	*NMDAR1*^{XC3}	0.968 (±0.027)	38.507 (±1.555)	40.442 (±2.586)	4.038 (±0.408)	10.00 (±0.308)	-64.621 (±1.368)	7	-
S6D	*NMDAR1*$^{-/-}$ +*GluRIIA*$^{-/-}$	*NMDAR1*^{XC3};*GluRIIA*^{SP16}	0.538 (±0.026)	32.277 (±1.762)	60.094 (±2.893)	2.558 (±0.281)	15.75 (±0.750)	-71.996 (±1.471)	4	<0.001 (***), <0.01 (**), <0.001 (***)
S6D	*NMDAR2*$^{-/-}$	*NMDAR2*^{YH7}	1.349 (±0.093)	37.346 (±1.990)	28.270 (±3.315)	3.291 (±0.689)	14.50 (±0.866)	-65.997 (±1.352)	4	-
S6D	*NMDAR2*$^{-/-}$ +*GluRIIA*$^{-/-}$	*NMDAR1*^{YH7};*GluRIIA*^{SP16}	0.523 (±0.011)	29.559 (±0.953)	56.582 (±2.114)	2.212 (±0.375)	21.25 (±0.629)	-70.517 (±1.497)	4	<0.001 (***), <0.05 (*), <0.001 (***)

Figure	Label	Genotypes	Quantal size (ΔF/F)	n	P Value (significance):
2F	wild type	*w;+;MHC>GCaMP6f/+*	0.020 (±0.00091)	12	-
2F	*GluRIIA*$^{-/-}$	*W;GluRIIA*^{SP16}*;MHC>GCaMP6f/+*	0.010 (±0.00026)	12	<0.0001 (****)
2F	G14>PV	*w;G14-GAL4/+;UAS-PV/MHC>GCaMP6f*	0.138 (±0.00061)	11	<0.0001 (****)
2F	*GluRIIA*^{Q615R}	*w;GluRIIA*^{Q615R}*;MHC>GCaMP6f/+*	0.010 (±0.00033)	12	<0.0001 (****)
2F	G14>PV+*GluRIIA*^{Q615R}	*w;GluRIIA*^{Q615R}*,G14-GAL4/GluRIIA*^{Q615R}*;UAS-PV/ MHC>GCaMP6f*	0.0077 ((±0.00034)	12	<0.0001 (****)
3B	MHC>GCaMP8f	*w;+;MHC>GCaMP8f/+*	0.0354 (±0.00672)	8	-
3B	+ OK319>BoNT-C	*w;OK319-GAL4/+;MHC>GCaMP8f/UAS-BoNT-C*	0.0013 (±0.00007)	8	<0.0001 (****)

Figure	Label	Genotypes	Quantal size (ΔF/F)	Frequency (Hz)	n	P Value (significance): Quantal size, Frequency
S5B	wild type	*w;+;MHC>GCaMP6f/+*	0.537 (±0.0145)	1.975 (±0.0943)	6	-
S5B	*GluRIIA*$^{-/-}$	*W;GluRIIA*^{SP16}*;MHC>GCaMP6f/+*	0.490 (±0.0158)	1.923 (±0.1251)	6	0.8513 (ns), 0.5505 (ns)

Figure	Label	Genotypes	Quantal size (ΔF/F)	Frequency (Hz)	n	P Value (significance): Quantal size, Frequency
S2WB	wild type	*w;+;MHC>GCaMP6f/+*	0.537 (±0.0145)	1.975 (±0.0943)	6	-
S2WB	*GluRIIA*$^{-/-}$	*W;GluRIIA*^{SP16}*;MHC>GCaMP6f/+*	0.490 (±0.0158)	1.923 (±0.1251)	6	0.8513 (ns), 0.5505 (ns)

Supplementary Table 2

REAGENT/RESOURCE	SOURCE	IDENTIFIER
**Antibodies**		
Mouse anti-GluRIIA (8B4D2)	Developmental Studies Hybridoma Bank (DSHB)	AB_528269
Mouse anti-DLG (4F3)	DSHB	AB_528203
Mouse anti-β-tubulin (E7)	DSHB	AB_2315513
Rabbit anti-parvalbumin	Thermo Fisher	Pa1-933
Rabbit anti-GluRIIB	(Perry et al., 2017)	
Rabbit anti-GluRIIC	(Goel and Dickman, 2018)	
Guinea pig anti-GluRIID	(Perry et al., 2017)	
Affinity purified Rabbit anti-pCaMKII	This study	
Affinity purified Guinea pig anti-CaMKII	This study	
Affinity purified Guinea pig anti-GluRIIA C-tail	This study	
Alexa Fluor 647 conjugated Goat anti-Horseradish Peroxidase	Jackson ImmunoResearch Laboratories	123-605-021
Alexa Fluor 488 conjugated secondary antibodies	Jackson ImmunoResearch Laboratories	706-545-148, 715-545-150, 711-545-152
Cy3-conjugated secondary antibodies	Jackson ImmunoResearch Laboratories	706-165-148, 715-165-150, 711-165-152
DyLight 405-conjucated secondary antibodies	Jackson ImmunoResearch Laboratories	706-475-148, 715-475-150
Horseradish Peroxidase conjugated secondary antibodies	Jackson ImmunoResearch Laboratories	115-035-003, 106-035-003
**Drosophila Strains**		
UAS-PV	This study	
UAS-GluRIIB	This study	
$GluRIIA^{Q615R}$	This study	
$GluRIIA^{\Delta C20}$	This study	
$GluRIIA^{\Delta C6}$	This study	
$GluRIIA^{QR\Delta C19}$	This study	
$UAS\text{-}GluRIIB^{IIAtail}$	This study	
SynapGCaMP8f	This study	
$NMDAR1^{XC3}$	(Li et al., 2021)	
$NMDAR2^{YH7}$	(Li et al., 2021)	
G14-GAL4	(Aberle et al., 2002)	
MHC-GAL4	(Schuster et al., 1996)	
OK319-GAL4	(Sweeney et al., 1995)	
MHC-CD8-GCaMP6f-Sh	(Newman et al., 2017)	
$GluRIIA^{SP22}$	(Diantonio et al., 1999)	
$UAS\text{-}CaMKII^{Ntide}$	(Chang et al., 1998)	
$GluRIIA^{SP16}$	(Petersen et al., 1997)	
UAS-BoNT-C	(Ramesh et al., 2021)	
NMDAR1-T2A-GAL4	(Kondo et al., 2020)	
NMDAR2-T2A-GAL4	(Kondo et al., 2020)	
$UAS\text{-}CaMKII^{T287D}$	Bloomington Drosophila Stock Center (BDSC)	29665
$UAS\text{-}CaMKII^{T287A}$	BDSC	29663
$UAS\text{-}CaMKII^{Ala}$	BDSC	29666

UAS-CaMKII	BDSC	29662
UAS-CaMKII^{RNAi}	BDSC	35330
nos-Cas9	BDSC	78782
Tub-PBac	BDSC	8283
attP2	BDSC	8622
w^{1118}	BDSC	5905
UAS-GluRIIA^{M614R}	BDSC	64256
Df(2L)cl^{h4}	BDSC	6304
**Construct**		
pACU	Addgene	58373
pACU2	Addgene	31223
pAC-U63-tgRNA	Addgene	112811
pHD-DsRed	Addgene	51434
**Chemical**		
BAPTA-AM	Abcam	120503
**Software and Algorithms**		
NIS Elements software	Nikon	4.51.01
Axon pCLAMP Clampfit	Molecular Devices	10.7
MiniAnalysis	Synaptosoft	6.0.3
Excel	Microsoft	2016
GraphPad Prism	GraphPad	8.0.1
Jupyter Notebook	Anaconda	6.0.1
ImageJ (Fiji)	(Rueden et al., 2017)	

Supplementary References

Aberle, H., Haghighi, A.P., Fetter, R.D., McCabe, B.D., Magalhães, T.R., and Goodman, C.S. (2002). Wishful thinking encodes a BMP type II receptor that regulates synaptic growth in Drosophila. Neuron *33*, 545–558. https://doi.org/10.1016/S0896-6273(02)00589-5.

Chang, B.H., Mukherji, S., and Soderling, T.R. (1998). Characterization of a calmodulin kinase II inhibitor protein in brain. Proceedings of the National Academy of Sciences *95*. .

Diantonio, A., Petersen, S.A., Heckmann, M., and Goodman, C.S. (1999). Glutamate receptor expression regulates quantal size and quantal content at the Drosophila neuromuscular junction. Journal of Neuroscience *19*. https://doi.org/10.1523/jneurosci.19-08-03023.1999.

Goel, P., and Dickman, D. (2018). Distinct homeostatic modulations stabilize reduced postsynaptic receptivity in response to presynaptic DLK signaling. Nature Communications *9*, 1–14. https://doi.org/10.1038/s41467-018-04270-0.

Kondo, S., Takahashi, T., Yamagata, N., Imanishi, Y., Katow, H., Hiramatsu, S., Lynn, K., Abe, A., Kumaraswamy, A., and Tanimoto, H. (2020). Neurochemical Organization of the Drosophila Brain Visualized by Endogenously Tagged Neurotransmitter Receptors. Cell Reports *30*, 284-297.e5. https://doi.org/10.1016/J.CELREP.2019.12.018/ATTACHMENT/EDF3DE7F-C3FD-42D5-83CD-87E341B7BBA9/MMC2.XLSX.

Li, X., Chien, C., Han, Y., Sun, Z., Chen, X., and Dickman, D. (2021). Autocrine inhibition by a glutamate-gated chloride channel mediates presynaptic homeostatic depression. Science Advances *7*, 1215. https://doi.org/10.1126/SCIADV.ABJ1215/SUPPL_FILE/SCIADV.ABJ1215_SM.PDF.

Newman, Z.L., Hoagland, A., Aghi, K., Worden, K., Levy, S.L., Son, J.H., Lee, L.P., and Isacoff, E.Y. (2017). Input-Specific Plasticity and Homeostasis at the Drosophila Larval Neuromuscular Junction. Neuron *93*, 1388-1404.e10. https://doi.org/10.1016/j.neuron.2017.02.028.

Perry, S., Han, Y., Das, A., and Dickman, D. (2017). Homeostatic plasticity can be induced and expressed to restore synaptic strength at neuromuscular junctions undergoing ALS-related degeneration. Human Molecular Genetics *26*, 4153–4167. https://doi.org/10.1093/hmg/ddx304.

Petersen, S.A., Fetter, R.D., Noordermeer, J.N., Goodman, C.S., and DiAntonio, A. (1997). Genetic analysis of glutamate receptors in drosophila reveals a retrograde signal regulating presynaptic transmitter release. Neuron *19*, 1237–1248. https://doi.org/10.1016/S0896-6273(00)80415-8.

Ramesh, N., Escher, M.J.F., Mampell, M.M., Böhme, M.A., Götz, T.W.B., Goel, P., Matkovic, T., Petzoldt, A.G., Dickman, D., and Sigrist, S.J. (2021). Antagonistic interactions between two Neuroligins coordinate pre- and postsynaptic assembly. Current Biology *31*, 1711-1725.e5. https://doi.org/10.1016/J.CUB.2021.01.093.

Rueden, C.T., Schindelin, J., Hiner, M.C., DeZonia, B.E., Walter, A.E., Arena, E.T., and Eliceiri, K.W. (2017). ImageJ2: ImageJ for the next generation of scientific image data. BMC Bioinformatics *18*, 1–26. https://doi.org/10.1186/s12859-017-1934-z.

Schuster, C.M., Davis, G.W., Fetter, R.D., and Goodman, C.S. (1996). Genetic dissection of structural and functional components of synaptic plasticity. I. Fasciclin II controls synaptic stabilization and growth. Neuron *17*, 641–654. https://doi.org/10.1016/S0896-6273(00)80197-X.

Sweeney, S.T., Broadie, K., Keane, J., Niemann, H., and O'Kane, C.J. (1995). Targeted expression of tetanus toxin light chain in Drosophila specifically eliminates synaptic transmission and causes behavioral defects. Neuron *14*, 341–351. https://doi.org/10.1016/0896-6273(95)90290-2.
