# Supplementary material for: A glutamate receptor C-tail recruits CaMKII to suppress retrograde homeostatic signaling
Source: Nat Commun. 2022 Dec 10;13:7656. doi: 10.1038/s41467-022-35417-9 (PMC9741633; doi:10.1038/s41467-022-35417-9)
Supplement: Supplementary file 3 — Reporting Summary [file 41467_2022_35417_MOESM3_ESM.pdf]

nature portfolio

Corresponding author(s): Dion Dickman

Last updated by author(s): Nov 28, 2022

Reporting Summary

Nature Portfolio wishes to improve the reproducibility of the work that we publish. This form provides structure for consistency and transparency in reporting. For further information on Nature Portfolio policies, see our Editorial Policies and the Editorial Policy Checklist.

Statistics

For all statistical analyses, confirm that the following items are present in the figure legend, table legend, main text, or Methods section.

n/a	Confirmed	
☐	☒	The exact sample size (n) for each experimental group/condition, given as a discrete number and unit of measurement
☐	☒	A statement on whether measurements were taken from distinct samples or whether the same sample was measured repeatedly
☐	☒	The statistical test(s) used AND whether they are one- or two-sided<br>*Only common tests should be described solely by name; describe more complex techniques in the Methods section.*
☐	☒	A description of all covariates tested
☐	☒	A description of any assumptions or corrections, such as tests of normality and adjustment for multiple comparisons
☐	☒	A full description of the statistical parameters including central tendency (e.g. means) or other basic estimates (e.g. regression coefficient) AND variation (e.g. standard deviation) or associated estimates of uncertainty (e.g. confidence intervals)
☐	☒	For null hypothesis testing, the test statistic (e.g. F, t, r) with confidence intervals, effect sizes, degrees of freedom and P value noted<br>*Give P values as exact values whenever suitable.*
☒	☐	For Bayesian analysis, information on the choice of priors and Markov chain Monte Carlo settings
☒	☐	For hierarchical and complex designs, identification of the appropriate level for tests and full reporting of outcomes
☒	☐	Estimates of effect sizes (e.g. Cohen's d, Pearson's r), indicating how they were calculated

Our web collection on statistics for biologists contains articles on many of the points above.

Software and code

Policy information about availability of computer code

Data collection	Axon pClamp Clampfit 10.7 (Molecular Divices), NIS Elements software (4.51.01)
Data analysis	MiniAnalysis 6.0.3 (Synaptosoft), Excel 2016 (Microsoft), GraphPad Prism 8.0.1 (GraphPad Software), NIS Elements software General Analysis toolkit 4.51.01, ImageJ (Fiji), Jupyter Notebook 6.0.1 (Anaconda)

For manuscripts utilizing custom algorithms or software that are central to the research but not yet described in published literature, software must be made available to editors and reviewers. We strongly encourage code deposition in a community repository (e.g. GitHub). See the Nature Portfolio guidelines for submitting code & software for further information.

Data

Policy information about availability of data

All manuscripts must include a data availability statement. This statement should provide the following information, where applicable:

- Accession codes, unique identifiers, or web links for publicly available datasets
- A description of any restrictions on data availability
- For clinical datasets or third party data, please ensure that the statement adheres to our policy

The authors declare that the data supporting the findings of this study are available within the paper, the Source Data file, and the Supplementary Information and Supplementary Data files.

Human research participants

Policy information about studies involving human research participants and Sex and Gender in Research.

Reporting on sex and gender	N/A
Population characteristics	N/A
Recruitment	N/A
Ethics oversight	N/A

Note that full information on the approval of the study protocol must also be provided in the manuscript.

Field-specific reporting

Please select the one below that is the best fit for your research. If you are not sure, read the appropriate sections before making your selection.

☒ Life sciences ☐ Behavioural & social sciences ☐ Ecological, evolutionary & environmental sciences

For a reference copy of the document with all sections, see nature.com/documents/nr-reporting-summary-flat.pdf

Life sciences study design

All studies must disclose on these points even when the disclosure is negative.

Sample size	Sample sizes were chosen as established in the field; sizes are typically over 10 for each dataset.
Data exclusions	No data exclusions were used in this study.
Replication	The reproducibility of experiments can be found in the relevant figure legend in this paper and supplemental figure legends.
Randomization	Randomization was not used in this study.
Blinding	Experimentalists were blinded from data acquisition through analysis.

Reporting for specific materials, systems and methods

We require information from authors about some types of materials, experimental systems and methods used in many studies. Here, indicate whether each material, system or method listed is relevant to your study. If you are not sure if a list item applies to your research, read the appropriate section before selecting a response.

Materials & experimental systems

n/a	Involved in the study	
☐	☒	Antibodies
☒	☐	Eukaryotic cell lines
☒	☐	Palaeontology and archaeology
☐	☒	Animals and other organisms
☒	☐	Clinical data
☒	☐	Dual use research of concern

Methods

n/a	Involved in the study	
☒	☐	ChIP-seq
☒	☐	Flow cytometry
☒	☐	MRI-based neuroimaging

Antibodies

Antibodies used	Presented in Key Resources Table (mouse anti-GluRIIA, -DLG, -Beta tubulin; Rabbit anti-GluRIIB, -GluRIIC, -pCaMKII; Guinea Pig anti-GluRIID, CaMKII, GluRIIA-Ctail. Conjugated secondary antibodies detailed).
Validation	Testing of antibodies on mutants, immunoblots, and genetic manipulations to confirm established behavior (detailed in Supplementary Figures 1, 2, and 3). Secondary antibodies were confirmed by no primary controls, and tests on species-specific immunostaining behavior.

Animals and other research organisms

Policy information about studies involving animals; ARRIVE guidelines recommended for reporting animal research, and Sex and Gender in Research

Laboratory animals	Strains of Drosophila melanogaster were used in third-instar stages (48-72 hours after egg lay). Strains are detailed in Key Resources table (w1118, UAS-PV, UAS-GluRIIB, GluRIIA.Q615R, GluRIIA.dC20, GluRIIA.dC6, GluRIIA.QRdC19, UAS-GluRIIB.IIAtail, SynapGCaMP8f, NMDAR1.XC3, NMDAR2.YH7, G14-Gal4, MHC-Gal4, OK319-Gal4, SynapGCaMP6f, GluRIIA.SP22, UAS-CaMKII.Ntide, GluRIIA.SP16, UAS-BoNT-C, NMDAR1-T2A-Gal4, NMDAR2-T2A-Gal4, UAS-CaMKII.T287D, UAS-CaMKII.T287A, UAS-CaMKII.Ala, UAS-CaMKII, UAS-CaMKII.RNAi, nos-Cas9, Tub-PBac, attP2, UAS-GluRIIA.M614R, Df(2L)cl.h4)
Wild animals	This study did not involve wild animals.
Reporting on sex	Animals of both sexes were used in this study.
Field-collected samples	This study did not involve animals collected from the field.
Ethics oversight	No ethical approval was required for this study due to the exclusive use of invertebrate animals.

Note that full information on the approval of the study protocol must also be provided in the manuscript.
